# Challenges in the care for consanguineous couples: an exploratory interview study among general practitioners and midwives

**DOI:** 10.1186/1471-2296-13-105

**Published:** 2012-10-26

**Authors:** Marieke E Teeuw, Anouk Hagelaar, Leo P ten Kate, Martina C Cornel, Lidewij Henneman

**Affiliations:** 1 Department of Clinical Genetics, Section Community Genetics, EMGO Institute for Health and Care Research, VU University Medical Center, Amsterdam, The Netherlands; 2 Department of Public and Occupational Health, EMGO Institute for Health and Care Research, VU University Medical Center, Amsterdam, The Netherlands

**Keywords:** Consanguinity, Primary care, Risk communication, Cultural barriers, Preconception and prenatal care

## Abstract

**Background:**

It is often suggested that an effort must be made to increase awareness among consanguineous couples of their reproductive risk, and to refer them for genetic counseling if needed. Primary care professionals are considered most appropriate for addressing the subject and identifying couples at risk during consultations in their practice. This Dutch study aims to explore the experiences, attitudes and beliefs of such professionals regarding their care for consanguineous couples.

**Methods:**

Sixteen semi-structured interviews were conducted with midwives and general practitioners.

**Results:**

Although most primary care professionals considered it their task to inform couples about the risks of consanguinity, during consultations the topic was generally only briefly touched upon and quickly abandoned. Important reasons for this were professionals’ beliefs about religious and social values of couples, their low perception of the couples’ reproductive risk and expected limited feasibility of referral. Feelings of embarrassment regarding addressing consanguinity did not seem to play a significant role.

**Conclusions:**

Primary care professional beliefs about their clients’ religious and social values, their attitudes toward the risk, and perceived limited options for referral seem to conflict with the professional norm to address the topic of consanguinity.

## Background

Consanguineous marriages, in practice usually defined as unions between second cousins or partners more closely related, are common in North Africa, the Middle East and certain parts of Asia, with prevalence rates of more than 20% [1]. With the migration of groups from regions where consanguinity is a common practice, the prevalence of consanguineous marriages increased in Western countries. Rates in migrant populations are sometimes even higher than in the country of origin [2]. In the Netherlands consanguineous marriages concern mostly couples of Turkish and Moroccan origin.

It is well known that a higher risk of congenital disorders is present in children of consanguineous parents, with a reported average increased risk of 1.7 – 2.8% for the offspring of first-cousin couples over the baseline 2-3% risk in the general population [3]. Existing recommendations on consanguinity focus on the identification of couples that are at increased risk and offering them genetic counseling, preferably before conception [4-6]. The excess risk, however, is most marked for rare and often severe diseases, which can be hard to predict in the preconception phase, as often these diseases have not previously occurred in the family [7]. The most powerful tool available is a comprehensive family medical assessment, as this can provide clues about the couple’s additional risk of giving birth to a child with a genetic condition that has already occurred in the family, and the possibility of genetic testing. Taking a thorough family history, however, includes drawing a pedigree, and asking a comprehensive list of questions that requires at least some insight in genetics [4], while others have argued that an even more robust approach is needed [8]. Other possibilities which are sometimes available to consanguineous couples are genetic carrier screening based on ethnicity, or, when pregnant, an advanced ultrasound for detecting fetal anomalies [4].

In many countries, primary care professionals, such as general practitioners (GPs) or family physicians and midwives are the professionals who provide preconception and prenatal care, and can thus play an important role in the prospective identification of consanguineous couples at increased risk [5,9]. In the Netherlands, GPs and midwives are gatekeepers for specialist care such as clinical genetics. GPs see their clients in the preconception- and prenatal phases, although only a small percentage still provide prenatal care and few clients may have specific questions on consanguinity. Midwives generally see pregnant women early in their pregnancy, and some offer consultations for couples for preconception advice, although not on a regular basis, and also not (yet) reimbursed. A national program for preconception care is lacking at this stage.

The professional organizations of GPs, midwives, gynecologists and clinical geneticists have, however, different practice guidelines in place on how and when consanguinity should be dealt with. Some advise referral in the case of consanguinity, while others claim referral is only of importance in the case of a positive family history [6,10-12].

A Dutch study showed that only 29% of GPs reported discussing consanguinity with patients planning a pregnancy, and 67% addressed hereditary or congenital disorders in the family [9]. An earlier American study showed major differences in genetic services offered to consanguineous couples by clinical geneticists and genetic counselors [13]. Moreover, the total number of genetic counseling consultations for people from migrant populations is generally low [14,15]. It is suggested that lack of genetic knowledge as well as racialized and stereotypical perspectives of health care professionals are contributing to these low numbers [16].

In Western societies, consanguinity often evokes controversial reactions and is considered to be strongly associated with physical and mental problems in offspring [17,18]. The dominant Western attitude toward consanguinity might create barriers when it comes to addressing the topic in a health care setting [19,20]. Genetic issues are known to be regarded as difficult by primary health care workers and a deficiency of genetic knowledge in general has been shown [13,21,22]. In a recent focus group study assessing genetic educational needs, primary care professionals expressed their concern regarding being unqualified to deal with ethical dilemmas related to genetics, including consanguinity [23].

The situation outlined above raises the question to what extent consanguineous couples are identified in primary care practice, whether and how the topic of increased reproductive risk is addressed, and which factors influence this process. To investigate this, we conducted exploratory semi-structured interviews with GPs and midwives. The primary care workers’ practices, beliefs and attitudes on which we focus in our study will contribute to a better comprehension of the process of care, as well as suggest strategies for improvement of care with regard to the reproductive risk for consanguineous couples.

## Methods

### Study design and participants

We conducted semi-structured interviews with midwives and GPs. For the recruitment of professionals, purposive sampling was used. Sampling was focused on practices based in areas with a medium- to high prevalence of populations in which consanguinity is common. Midwives and GPs who were part of the researchers’ professional network were invited, as well as professionals listed as practicing in the corresponding target areas. The invitation by letter or e-mail mentioned that the purpose of the study was to explore how consanguinity was dealt with in primary practice. In the case of non-response, follow-up was done by telephone. Participants were offered a booklet on genetics in primary care as an incentive. Thirteen midwives were approached directly, three of whom declined to participate due to time constraints. Twelve GPs were approached directly, six of whom agreed to participate. Fifty other GPs were approached by e-mail by the research coordinator of a GPs network, none of whom responded positively to the request for participation. In these cases, non-response was not followed-up. Reasons given by GPs for not participating were lack of time and having no experience with consanguineous couples. Ultimately, ten midwives and six GPs were interviewed. All professionals were of Dutch origin, except for one midwife who was from Moroccan descent. All but one professional had experience with consanguineous couples; some on an occasional basis, but most claimed to see consanguineous couples regularly. Characteristics of participants are shown in Table 1.

**Table 1:** Characteristics of participating primary care professionals (n = 16)

	**Midwives n = 10**	**General Practitioners (GPs) n = 6**^***a***^
**Gender**		
Female	10	3
Male		3
**Work experience**		
<5 years	3	1
5 – 10 years	2	2
>10 years	6	3
Average work experience in years (sd)	12.1 (2.3 – 21.9)	15.4 (5.3 – 24.5)

^*a*^ None of the interviewed GPs were providers of prenatal care.

### Interview guide and procedure

Elements of Triandis’ theory of attitude-behavior relations were used to develop the interview guide. The Triandis model describes how behavior is influenced by affect, attitude and social norms on the one hand, and habits and facilitating factors on the other [24]. This model is particularly useful for culturally sensitive situations [25]. The interview guide contained the following subjects: (i) if and how the topic of consanguinity was addressed, (ii) attitudes and beliefs towards addressing consanguinity and risk, and experiences with it, (iii) barriers that are encountered when addressing the topic, (iv) personal beliefs towards consanguinity.

The interviews were conducted by two researchers (AH and MT). The interviews took place in the practices or at the homes of the professionals and lasted between 30 to 70 minutes. Prior to each interview, all participants gave informed consent. The interviews were recorded with audio equipment. The study protocol was approved by the Medical Ethical Committee of the VU University Medical Center in Amsterdam.

### Data preparation and analyses

All interviews were transcribed and content analysis was done using the software program Atlas.ti, version 5.2. The first three interviews were independently coded by three researchers (AH, MT, LH). Coding was compared for reliability and consensus was reached. The codings were subsequently clustered into key themes and sub-themes. All remaining interviews were coded by two researchers (AH, MT) and discussed until agreement was reached (AH,MT,LH). For the present report interview statements were translated from Dutch. Characteristics of participants are given in brackets, indicating the profession (midwife (MW) or general practitioner (GP)), number of interview (1–16), and how often they report seeing consanguineous couples (regularly/occasionally/never).

## Results

These results are reported in adherence to the RATS guidelines for qualitative research [26].

Most professionals considered it very important that consanguineous couples are aware of their increased reproductive risk associated with consanguinity and almost all agreed that it is in particular a task for their own profession to address the topic. Only one GP did not believe this to be his task, and suggested that schools and municipal health centers are more appropriate places for informing the target group. However, despite their normative beliefs, the professionals also reported that in their own practices the topic of consanguinity and reproductive risk was generally only briefly discussed, if raised at all, and quickly abandoned.

The most important themes that emerged from the interviews regarding factors that influence whether and how the topic of consanguinity and risk is addressed were: a) Beliefs about consanguineous couples’ religious and social values; b) Attitude toward the nature of the risk; c) Attitude toward options for referral and standards of care; d) Topic’s sensitivity; e) Setting and timing of consultation.

### Beliefs about consanguineous couples’ religious and social values

The majority of the professionals felt that information about the risks of consanguinity is not welcomed by their clients, resulting in the topic being addressed only briefly during consultations. The general feeling was that though most couples have heard about the existence of an increased risk, their clients do not appear to be interested in receiving more information. Both midwives and GPs asserted that when trying to explain more about the topic, it is often ended abruptly, due to a defensive or negative response from the clients. The professionals believed that this type of response was generally the result of religious motives:

“They say: ‘if we have a child that’s not right, then it’s God’s will and we don’t want to know beforehand and certainly don’t want to have it examined [prenatally]’.” (MW7, occasionally)."

As a consequence some professionals felt that it is easier for their clients not to know or to deny the risk, because the consequences following from the risk information – like genetic testing and possible termination of pregnancy – are not considered acceptable. Another important factor mentioned by some professionals was the fact that the opinion of the community and family of their clients plays a very important role.

“I notice that in the case of young women… that there is so much pressure caused by what the family thinks of the partner. […]They don’t look at the person himself, but where he is from and whether they know him. And then it often comes down to a first or second cousin. Information about risk does not reach [these women], because the partner choice is primarily influenced by the family.” (GP14, regularly)."

Moreover, in the experience of some of the professionals, the tradition of consanguineous marriage in the immigrant communities often coexists with a poor command of the Dutch language, and therefore they often refrain from serious attempts to inform their clients.

“Because those people, who have this problem [consanguinity] often don’t speak any Dutch, or just a little bit. The language barrier obstructs to a great extent.” (MW3, regularly)."

As a result of the beliefs resulting from these previous experiences, professionals sometimes do not even raise the topic during new consultations. They mentioned that they do not want to burden their clients with detailed information if they know their culture will not allow them to contemplate taking action.

“It’s a very different dialogue with religious Muslims. […] I use three sentences [to address the risk], so I leave information out, […] because I don’t want to upset people without good reason.” (MW1, regularly)."

Some of the professionals have noticed that the younger generation of Turkish and Moroccan women who were raised and educated in the Netherlands seem increasingly open to discuss the topic of consanguinity, especially when coming from a female care professional. Also, options like prenatal diagnosis and abortion can be discussed with the younger generation, however, when it comes to the possibility of referral for further diagnostic procedures, the professionals mentioned that their clients eventually do not take up on the offer.

### Attitude toward the nature of the risk

Although almost all professionals admitted having limited knowledge on the topic, they seemed to be aware of the increased risk for consanguineous couples to have a child with a congenital disorder. The general feeling among them was that the risk was not very high. The fact that the risk was perceived to be rather small also influences to what extent the professionals try to inform their clients.

“Usually I leave [the information] at this, because, when the [wedding] plans are made, in my opinion it’s not very useful telling complex stories about the risks of congenital disorders. […] The risk is increased, but that’s on a population level. On an individual level these types of marriages are often without any problems.” (GP10, regularly)."

Half of the professionals mentioned having seen affected children being born in consanguineous relationships, though most mentioned that they were not sure if that was more often than in non-consanguineous relationships. Few professionals mentioned that they believed consanguinity entails a high risk and some felt the increase in risk was a subject of some debate among experts, and that there were no exact facts to communicate to clients. Some professionals mentioned spontaneously which diseases they thought the increased risk entails. Blood disorders, congenital heart disorders, spina bifida, physical and mental retardation, Down syndrome, blindness and deafness were among the diseases mentioned, not all of which are associated with consanguinity.

### Attitude toward options for referral and standards of care

Neither GPs nor midwives were aware of existing standards of care on consanguinity for their profession. Some mentioned having guidelines for their own practice, sometimes in collaboration with the obstetrics department of a nearby hospital, which mostly only include the offer of an advanced prenatal ultrasound. The general feeling among midwives was that there is only a need to refer their clients to specialist care, like a clinical genetic center, when they seem to have a positive family history for relevant diseases.

“If several things have occurred in the family then alarm bells start ringing. Otherwise I keep [the information] very limited.” (MW11, regularly)."

Midwives also admitted being quite reluctant to refer their clients, because they believed they are not supposed to refer all consanguineous couples.

“Because I’m wondering whether the clinical geneticist is willing to counsel every [consanguineously married] pregnant woman completely, because I don’t think they do it, don’t want to, that seems impossible.” (MW3, regularly)."

GPs tend to refer their clients to a clinical genetics center or to an obstetrics department when the couple has already had an affected child or if there is a history of spontaneous abortions. The GPs also regarded a positive family history as an indication for referral when a couple – consanguineous or not – wishes to have a child. They saw consanguinity in these cases as an extra indication, but not a reason for referral in itself.

Several professionals emphasized that taking a good family history is difficult for them, because often people are not aware of diseases in their family, and because they themselves lack the knowledge to correctly assess the impact of certain genetic disorders. A need for more clarity on the topic, in addition to improving skills for taking a good family history, was expressed frequently by both GPs and midwives.

“It’s good, I think, if the information concerning consanguinity and the risk of having children, that it would be more clear in order to have more tools you can use in practice.” (MW2, occasionally)."

### Topic’s sensitivity

When asked about the feeling that talking about the consanguinity of their clients yields, none of the professionals reported any embarrassment. The professionals considered asking about the existence of consanguinity a necessary and, in the case of midwives, standard question in the context of their work and also argued that it was not a sensitive issue for the couples.

“In my experience the people themselves, a woman from Morocco or Turkey, for them it is less of a taboo […], so for them it’s very natural.” (MW2, occasionally)"

They deemed this question less difficult than, for example, a question about the sexual history or possible abuse of the pregnant woman. Only in situations where the couple had a history of an affected child, some professionals reported some unease and worry about being too paternalistic or even judgmental.

When asked about their personal ideas about consanguinity, most participants admitted they believed consanguinity to be inappropriate. Although the sensitivity of the topic was recognized by all, most reported that their experience as professionals has made them less judgmental.

“I don’t want to think of it as aberrant. I think if people love each other, yes, fine.” (MW11, regularly)."

Only two GPs mentioned, however, that they believed people should not marry a cousin. One of them was the only professional without reported experience with consanguineous couples:

“Inbreeding, inbreeding is never good.” (GP5, never)."

### Setting and timing of consultation

GPs and midwives reported that in almost all cases, the moment at which the topic of consanguinity and risk is raised, depends on the initiative of the professional.

Among midwives, asking a woman or couple if they are consanguineously married is often a standard question during an intake in early pregnancy. Among GPs, however, there is no standard setting where this question is asked, especially when they do not provide prenatal care, which is the case with the great majority of GPs. Raising the topic is always done in the context of another consultation, during which for example wedding plans are mentioned or a history of spontaneous abortions is discussed. Consequently, limited available time influences the intentions to address the topic.

“People often come with lots of problems at the same time, and [the risks associated with consanguinity] get the least priority, if they themselves don’t start talking about it.” (GP10, regularly)."

The timing of giving information about consanguinity and risk was also seen as a crucial factor. Professionals felt it best to inform people before wedding plans arise or otherwise before the first pregnancy, because of the beliefs already described.

“When a cousin couple comes when they’re already pregnant there is nothing left to do.” (MW4, regularly)."

## Discussion

This study aimed to gain insight into the experiences, attitudes and beliefs of midwives and GPs when dealing with consanguineous couples, as they are in a position to play an important role in identifying these couples, and to bring up the topic of associated risk for offspring. The main finding of the study is that there seem to be conflicting issues at play in the professionals’ practices. Although almost all professionals agreed that addressing the topic of consanguinity is a task for primary care, it is mostly not discussed thoroughly between them and their clients, and when it is discussed, it is quickly abandoned.

According to the professionals, a major obstacle consists of a defensive or negative attitude of the clients, because they feel their clients have already committed themselves to their marriage and are religiously barred from considering other reproductive options. Previous studies in the Netherlands among migrant populations, report that although preconceptional testing is generally evaluated as positive [27,28], there is more reluctance toward prenatal testing and termination of pregnancy in some populations [27,29]. It has been argued, however, that the Islamic religion should not be taken as a proxy for women’s attitudes either for or against termination of pregnancy, because they are influenced by various other factors, and faith is not so much prescriptive as well as important in a broader moral context [30,31]. At the same time, some professionals in our study felt there is a shift noticeable, especially in young females, who appear to be more and more open to discussing this topic and the consequences of the risk. This finding is in line with international literature reporting an increasing proportion of people from communities with a tradition of consanguinity seeking genetic counseling [32]. The openness of younger generations may generate possibilities for informing specific groups of (female) migrants, in which other issues related to pregnancy care are also discussed, or for the use of social media.

Another important obstacle in discussing consanguinity is the fact that the professionals feel somewhat insecure about what the risk for consanguineous couples entails. Furthermore, they believed the risk to be rather low and limited to a minority of the couples. Although professionals claim to be mostly capable of referring people when necessary, they also regard the options for referral rather nontransparent and limited.

When the reproductive risk associated with consanguinity is perceived as low, when clear standards of care are lacking, and, at the same time, clients appear to be unresponsive, the fact that few referrals take place may not be surprising. On the one hand it is conceivable and correct that this topic should not be forced upon clients when they claim not to be interested, given the central position of the ethical principle of “autonomy” in genetic counseling [33]. On the other hand, with the reported language barriers in mind, as well as the fact that new clients are believed in advance to be not open to discussing the topic because of previous experiences of professionals, a vicious circle may be followed by continuous lack of awareness.

It was suggested in the literature that the Western taboo on consanguinity might play a role in bringing up the topic [20], but that was not found in this study, which could be caused by cultural differences between countries. None of the professionals reported that embarrassment prevented them from asking the question about the presence of consanguinity. Some admitted, however, experiencing some unease in stressing the association with risk to the offspring of couples.

Although this study was not designed to measure knowledge of the professionals, it appears that there is some room for improvement of knowledge and skills. The average risk was considered high by some and Down syndrome for example was mentioned frequently, while this risk is not increased in consanguineous couples [34]. The absence of a positive family history was also considered by several professionals as reassuring, while the reproductive risk can still be substantial. Participants also acknowledged difficulties when taking a family history. Given the international recommendations on this topic, one could question if this level of knowledge and skills in primary care is adequate when assessing the risk in an individual consanguineous couple [4,32], or whether a (short) consultation in a genetic clinic would be more appropriate.

Some limitations of our study should be noted. Respondents were purposefully sampled midwives and GPs, probably with a more than average interest in consanguinity. Furthermore, the face-to-face setting in which the interview took place might have caused the respondents to be somewhat cautious, given the sensitivity of the topic. An additional quantitative research approach is needed to enable generalization of our findings.

It is important to realize that GPs and midwives work in essentially different settings. Despite the fact that they are considered the professionals most likely to identify the couples in a preconception phase, GPs have no setting in which the topic is easily addressed. This is different for midwives whose standard intake procedure contains a question on consanguinity. Although the offer of preconception consultations exists, midwives primarily see these couples in the prenatal phase. Since the importance of timing was frequently mentioned by our professionals, the possibility to further develop preconception care should be included when developing future education and guidelines for primary care.

## Conclusions

This study clearly shows that the dynamics of the process of identifying and referring consanguineous couples in primary care is complex. Primary care professionals consider it their task to address the topic of consanguinity and risk during consultations. However, despite the fact that the taboo does not prevent them from raising it, the topic is also quickly set aside, because of the professionals’ belief that clients are not interested, as well as their perception that both risk and options for referral are limited.

Primary care professionals can be regarded as crucial actors in the health service infrastructure where consanguineous couples at high risk are identified and referred. The views posed by the primary care professionals seem to reflect the ambiguous practical guidelines that are in place. Earlier publications have also already described a need for consensus on how to approach consanguinity [5,13,32] and this study confirms these suggestions. Special attention should be focused on the setting and timing of this care. These efforts should be made sooner rather than later, since ongoing developments in genetic technology are expected to generate many opportunities for screening and testing in the near future, which can for example be used in preconception settings in the case of consanguinity [7,35]. With the extra risk for mostly rare monogenic diseases in their offspring, consanguineous couples will be eligible for whole genome approaches, but before this can be put into clinical practice, it needs to correspond with the desires and beliefs of the target population. Therefore, the culturally sensitive issues that appear to be playing an important role need to be addressed above all and an investigation of the target population seems to be essential at this point.

## Competing interests

The authors declare that they have no competing interests.

## Authors' contributions

All authors made a substantial contribution to the concept and design of the study. MT and AH carried out the interviews and performed the analysis. MT drafted the manuscript. LH supervised the study, participated in the analysis and helped to draft the manuscript. AH, LK and MC were involved in drafting and revising the manuscript. All authors read and approved of the manuscript.

## Pre-publication history

The pre-publication history for this paper can be accessed here:

http://www.biomedcentral.com/1471-2296/13/105/prepub
